# Influenza vaccination uptake among at‐risk patients in Switzerland—The potential of national claims data for surveillance

**DOI:** 10.1111/irv.13206

**Published:** 2023-10-13

**Authors:** Andreas Plate, Christophe Bagnoud, Thomas Rosemann, Oliver Senn, Stefania Di Gangi

**Affiliations:** ^1^ Institute of Primary Care University of Zurich and University Hospital Zurich Zurich Switzerland; ^2^ Groupe Mutuel Martigny Switzerland

**Keywords:** at‐risk patients, claims data, influenza, vaccination, vaccination uptake

## Abstract

**Background:**

Swiss national surveillance of influenza vaccination uptake rates (VURs) relies on self‐reported vaccination status. The aim of this study was to determine VURs among at‐risk patients, namely, patients ≥65 of age and adult patients with chronic diseases, using claims data, instead of self‐reported measures, to investigate factors of vaccine uptake, and to assess different methodological approaches to conduct vaccination surveillance.

**Methods:**

In this retrospective cross‐sectional analysis, we determined VURs in three influenza seasons (2015/2016–2017/2018). Medication, diagnosis, or medical services claims were used as triggers to identify patients. For the calculation of VURs in patients with chronic diseases, we identified those by triggers in the given season only (Model 1) and in the given and previous seasons (Model 2). Regression analysis was used to identify factors associated with vaccination status.

**Results:**

Data from 214,668 individual patients were analyzed. VURs over all seasons ranged from 18.4% to 19.8%. Most patients with chronic diseases were identified with the medication trigger, and we found no clinical significant differences in VURs comparing both models. Having a chronic disease, age, male gender, and regular health care provider visits were associated with increased odds of being vaccinated.

**Conclusions:**

VURs were below the recommended thresholds, and our analysis highlighted the need for efforts to increase VURs. We assessed the identification of chronic diseases by medication claims and the calculation of VURs based on data of the given season only as an effective approach to conduct vaccination surveillance. Claims data‐based surveillance may complete the national surveillance.

## 1 INTRODUCTION

In accordance to the World Health Organization (WHO) recommendations,^1^, ^2^ vaccination against seasonal influenza is recommended in Switzerland for population groups at increased risk for complications such as people ≥65 of age or with chronic diseases.^3^ The increased risk in these groups is driven by many factors, for example, the decline in immune function and a reduced vaccine effectiveness. Moreover, multimorbidity is associated with increased hospitalization rates or mortality in people ≥65 of age.^4^, ^5^ National vaccination uptake rates (VURs) are about 35% among elderly and patients with chronic diseases^6^ and thus far below the WHO recommended target of 75%.^7^, ^8^ Vaccination rates in Switzerland are in the middle range for Europe.^8^, ^9^ Influenza results in as many as 330,000 consultations and up to 5000 hospitalizations annually in Switzerland, and nationwide activities aim to increase vaccination rates.^10^, ^11^ The decline in vaccination uptake over the past years^11^ is thus a growing concern, highlighting the need for close monitoring.

Monitoring VURs is a cornerstone in the WHO global influenza strategy^1^, ^2^ and essential for effective interventions to increase VURs. Swiss national surveillance, as in the United States,^12^ relies on data obtained from health surveys.^11^, ^13^, ^14^ Self‐reports may overestimate true VURs^15^, ^16^ or not be appropriate for at‐risk populations with chronic diseases.^17^, ^18^ Reliable calculations of VURs, taking into account chronic diseases and based on routinely available data, are needed. Moreover, knowledge about factors associated with vaccination uptake is relevant to impact not only the public health burden associated with influenza^19^ but also holistic outcomes such as quality of life^20^ or health care costs.^21^, ^22^

The aim of our study was therefore to determine VURs among two risk groups: people ≥65 of age and adults with chronic diseases, using routinely available claims data. In addition, we explored factors associated with influenza vaccination uptake in Switzerland and assessed the claims data potential to conduct influenza vaccination surveillance.

## 2 METHODS

### 2.1 Study design and database

Anonymized data for this retrospective, repeated cross‐sectional analysis, from 08/2015 to 07/2018, were provided by the health insurance company Groupe Mutuel (GM)^23^ according to the below criteria. Due to internal policy regulation, GM delivered a random sample of 95% of identified patients with subsequent years' data as long as the patient was alive and insured at GM. Only claims from the national basic compulsory health insurance were included. Part of the incurred costs are paid by the patient as a deductible. Deductibles range between 300 and 2500 CHF per year. Influenza vaccination, for at‐risk population, is covered by the basic insurance after the deductible is met.

### 2.2 Inclusion and exclusion criteria

We analyzed data of patients aged ≥65 years or adult patients (aged ≥18 years) with a chronic disease qualifying for influenza vaccination^3^ (hereafter referred to as at‐risk patients). GM identified chronic diseases using four trigger types based on respective claims: two medication‐based triggers (pharmaceutical cost group [PCG] and WHO Anatomical Therapeutic Chemical [ATC] codes), one diagnosis‐based trigger (diagnosis‐related group [DRG]), and one medical services‐based trigger (procedure codes [tariff medical, TARMED]). Patients may be identified by a single or more triggers (Table S1). Trigger codes were chosen to identify as many risk patients as possible. In Switzerland, PCG codes are used to even out risk compensation between health insurance companies^24^; DRG codes identify diagnosis during in‐hospital treatment^25^; and TARMED codes are used for ambulatory medical services reimbursement.^26^ ATC codes were chosen for some groups of immunosuppressive drugs not included in the PCG system. We considered all chronic diseases subject to recommendation within the national immunization schedule and identifiable through claims data such as chronic heart, lung, liver, and kidney diseases, cerebral diseases, diabetes mellitus, lymphoma, leukemia or myeloma, autoimmune disease or drug immunosuppression, human immunodeficiency viruses (HIV), cancer, and transplantations.

### 2.3 Data description and definitions

GM provided the following patient information: year of birth (age in years was calculated at each season), gender (male and female), long‐term care: living in nursing home (yes/no), Swiss citizenship (yes/no), health insurance model (family doctor, network, or telemedicine call center as a gatekeeper; free choice model where patients have full freedom to receive treatments), deductible (Level 1: ≤500 Swiss francs [CHF]; Level 2: 501–1500 CHF; and Level 3: 1501–2500 CHF), area of residence (defined according to the Swiss cantons [cantons represent the member states of Switzerland] main speaking languages: French, German, and Italian), number of all active PCG (surrogate for the number of comorbidities), and number of general practitioners (GPs) and specialists visits (defined as categorical: 1–5, 6–10, and >10 consultations). Diseases of immunocompromised (ICP) group included cancer, HIV, autoimmune disease or drug immunosuppression, lymphoma, leukemia or myeloma, and transplantations. Influenza vaccinations were identified by ATC codes J07BB01–04 and each influenza season from August to July of the following year. The period, exceeding the clinical influenza season, was chosen to be able to include all patients' claims incurred during the clinical influenza season but recorded subsequently by the insurance. The study was reported in accordance with the STROBE (Strengthening the Reporting of Observational Studies in Epidemiology) checklist for cross‐sectional studies.^27^

### 2.4 Outcomes

The main outcome was the VUR in 2015/2016–2017/2018. It was defined, for each season, as the proportion of vaccinated patients over the total at‐risk patients, overall, and then stratified by type of risk. Secondary outcomes were potential factors associated with vaccination status. Finally, we determined the relative importance of the different identifying triggers and assessed two different claims data models on their potential to conduct vaccination surveillance. Model 1 was based on a given season data; Model 2 was based on all available data to determine the chronic disease status. For example, in Model 2, a patient retained the chronic heart disease diagnosis over all seasons, even if a corresponding trigger was present only in the first season and not in the following ones.

### 2.5 Statistical analysis

Baseline characteristics of patients in each season were described as number and percentage, *N* (%), for categorical or binary variables and as mean (standard deviation [SD], range [minimum–maximum]) for continuous variables. Available case analysis was performed. Type of risk patients and vaccination uptakes were reported as number and percentage, *N* (%).

A multivariable mixed logistic regression model was performed to identify factors associated with vaccination status. Random effects at the patient level accounted for repeated measurements within patients included in more than one season. Multivariable analysis factors were selected through a stepwise backward approach, starting from a full model including all variables described above, not correlated among them. Likelihood ratio testing, *p* value ≤ 0.20, was the criteria to exclude variables. Results were represented through an odds ratio (OR) plot and reported as OR (95% confidence interval [CI]).

The relative importance of each trigger, in each season and by comorbidity, was represented using Venn diagrams.

Agreement in chronic diseases identification between Model 1 and Model 2 was measured using the Cohen's κ coefficient.^28^ According to McHugh,^29^ agreement was strong and, for our purposes, acceptable, if Cohen's κ coefficient was in the range of 0.80–0.90, meaning agreement of 80%–90%. Cohen's κ coefficient above 0.90 meant almost perfect agreement, above 90%. Vaccination uptakes calculated with the two models for each risk group were compared using chi‐square test for two proportions, two‐sided. For all tests, *p* ≤ 0.05 was considered statistically significant. All analyses were carried out using statistical package R Version 4.1.0.^30^ In particular, for Venn diagrams, we used package ‘ggVennDiagram’.

### 2.6 Ethics

Research using anonymized and aggregated claims data did not fall under the Swiss Federal Act on Research involving Human Beings (Human Research Act) and no ethics approval was needed.

## 3 RESULTS

We included a total of 214,668 individual patients. Baseline characteristics are presented in Table 1. Proportion of identified chronic diseases are presented in Table 2.

**TABLE 1:** Baseline characteristics stratified by influenza season.

		Influenza season		
		2015/2016	2016/2017	2017/2018
Individual patients, *n*		167,607	177,172	182,429
Sex^a^	Female	83,230 (49.7)	88,261 (49.8)	91,225 (50.0)
Age, mean (SD, range [minimum–maximum])		67.40 (12.47, 18–106)	67.48 (12.39, 19–106)	67.53 (12.31, 18–106)
Language region	French	99,735 (59.5)	104,616 (59.0)	107,167 (58.7)
	German	60,467 (36.1)	64,729 (36.5)	67,118 (36.8)
	Italian	6673 (4.0)	7028 (4.0)	7230 (4.0)
	Abroad	732 (0.4)	799 (0.5)	914 (0.5)
Insurance model	Free choice	89,578 (53.4)	90,170 (50.9)	88,803 (48.7)
	Network	17,291 (10.3)	18,645 (10.5)	17,838 (9.8)
	Family medicine	40,979 (24.4)	45,944 (25.9)	50,220 (27.5)
	Telemedicine	19,759 (11.8)	22,413 (12.7)	25,568 (14.0)
Deductible level^b^	Level 1	140,526 (83.8)	148,155 (83.6)	151,996 (83.3)
	Level 2	18,533 (11.1)	19,130 (10.8)	19,291 (10.6)
	Level 3	8548 (5.1)	9887 (5.6)	11,142 (6.1)
Swiss nationality	Yes	123,578 (73.7)	130,536 (73.7)	133,919 (73.4)
Number of yearly GP consultations	No consultations	39,580 (23.6)	42,858 (24.2)	45,395 (24.9)
	1–5	73,942 (44.1)	79,753 (45.0)	83,315 (45.7)
	6–10	33,268 (19.8)	34,458 (19.4)	34,485 (18.9)
	>10	20,817 (12.4)	20,103 (11.3)	19,234 (10.5)
Number of yearly specialist consultations	No consultations	46,692 (27.9)	48,794 (27.5)	48,912 (26.8)
	1–5	75,099 (44.8)	79,388 (44.8)	81,746 (44.8)
	6–10	25,843 (15.4)	27,307 (15.4)	28,194 (15.5)
	>10	19,973 (11.9)	21,683 (12.2)	23,577 (12.9)
Living in nursing home		7586 (4.5)	8185 (4.6)	8319 (4.6)
Number of overall comorbidities (PCG) Mean (SD, range [minimum–maximum])		1.19 (1.21, 0–12)	1.26 (1.23, 0–9)	1.31 (1.25, 0–9)

*Note*: Unless otherwise specified, values are presented as absolute numbers and percentage.

Abbreviations: GP, general practitioner; PCG, pharmaceutical cost group; SD, standard deviation.

^a^ Sex was missing for five patients.

^b^ ≤500 (Level 1), 501–1500 (Level 2), and 1501–2500 (Level 3) CHF, 1 CHF = 1.0824 USD (01/01/2023).

**TABLE 2:** Overview of the at‐risk patients stratified by influenza season.

	Influenza season		
	2015/2016	2016/2017	2017/2018
Individual patients	167,607	177,172	182,429
Type of risk			
Age ≥ 65 without chronic disease	93,664 (55.9)	98,247 (55.5)	97,727 (53.6)
Age ≥ 65 independent of chronic disease	128,665 (76.8)	135,977 (76.7)	138,558 (76.0)
Chronic disease and age ≥ 65	35,001 (20.9)	37,730 (21.3)	40,831 (22.4)
Chronic disease and age < 65	38,942 (23.2)	41,195 (23.3)	43,871 (24.0)
Chronic disease independent of age	73,943 (44.1)	78,925 (44.5)	84,702 (46.4)
Chronic diseases			
Chronic heart diseases	12,476 (7.4)	13,175 (7.4)	13,665 (7.5)
Chronic lung diseases	13,751 (8.2)	14,962 (8.4)	15,270 (8.4)
Diabetes mellitus	24,188 (14.4)	26,164 (14.7)	30,257 (16.6)
Cancer	7167 (4.3)	7674 (4.3)	8453 (4.6)
Chronic kidney diseases	529 (0.3)	568 (0.3)	559 (0.3)
HIV	2114 (1.3)	2225 (1.3)	2214 (1.2)
Cerebral diseases	3831 (2.3)	4073 (2.3)	3929 (2.2)
Chronic liver diseases	210 (0.1)	166 (0.1)	312 (0.2)
Autoimmune disease or drug immunosuppression	17,826 (10.6)	19,206 (10.8)	20,493 (11.2)
Lymphoma, leukemia, or myeloma	186 (0.1)	184 (0.1)	299 (0.2)
Transplantations	821 (0.5)	897 (0.5)	882 (0.5)

*Note*: Values are presented as absolute numbers and percentage.

Abbreviation: HIV, human immunodeficiency viruses.

### 3.1 Vaccination uptake rates

Overall influenza VURs among at‐risk patients were 18.4%–19.8% (Table 3). Stratified by the present risk factor, the highest VURs in patients ≥65 of age with a chronic disease (31%–33.8%) and the lowest VURs in patients <65 of age with a chronic disease (14.2%–14.9%) were observed. VURs in patient groups with specific chronic diseases revealed the highest VURs in patients with HIV (39.3%–36.2%) and the lowest VURs in patients with chronic liver diseases (14.8%–17.9%). Patients in long‐term care had VURs of 38.4%–41.1%.

**TABLE 3:** Influenza vaccination uptakes stratified by influenza season, type of risk, chronic diseases, and long‐term care.

	Influenza season		
	2015/2016	2016/2017	2017/2018
Patients vaccinated	30,815 (18.4)	33,351 (18.8)	36,078 (19.8)
Type of risk			
Age ≥ 65 without chronic disease	14,412 (15.4)	15,232 (15.5)	15,729 (16.1)
Age ≥ 65 independent of chronic disease	25,268 (19.6)	27,345 (20.1)	29,546 (21.3)
Chronic disease and age ≥ 65	10,856 (31.0)	12,113 (32.1)	13,817 (33.8)
Chronic disease and age < 65	5547 (14.2)	6006 (14.6)	6532 (14.9)
Chronic disease independent of age	16,403 (22.2)	18,119 (23.0)	20,349 (24.0)
Chronic diseases			
Chronic heart diseases	3489 (28.0)	3735 (28.3)	4051 (29.6)
Chronic lung diseases	3460 (25.2)	3884 (26.0)	4234 (27.7)
Diabetes mellitus	5087 (21.0)	5645 (21.6)	6755 (22.3)
Cancer	1394 (19.5)	1662 (21.7)	1978 (23.4)
Chronic kidney diseases	142 (26.8)	183 (32.2)	183 (32.7)
HIV	830 (39.3)	865 (38.9)	802 (36.2)
Cerebral diseases	577 (15.1)	680 (16.7)	720 (18.3)
Chronic liver diseases	31 (14.8)	28 (16.9)	56 (17.9)
Autoimmune disease or drug immunosuppression	3543 (19.9)	4027 (21.0)	4675 (22.8)
Lymphoma, leukemia, or myeloma	41 (22.0)	49 (26.6)	77 (25.8)
Transplantations	317 (38.6)	318 (35.5)	316 (35.8)
Long‐term care			
Living in nursing home	2910 (38.4)	3277 (40.0)	3417 (41.1)

*Note*: Values are presented as absolute numbers and percentage.

Abbreviation: HIV, human immunodeficiency viruses.

### 3.2 Factors associated with vaccination status

**FIGURE 1:**
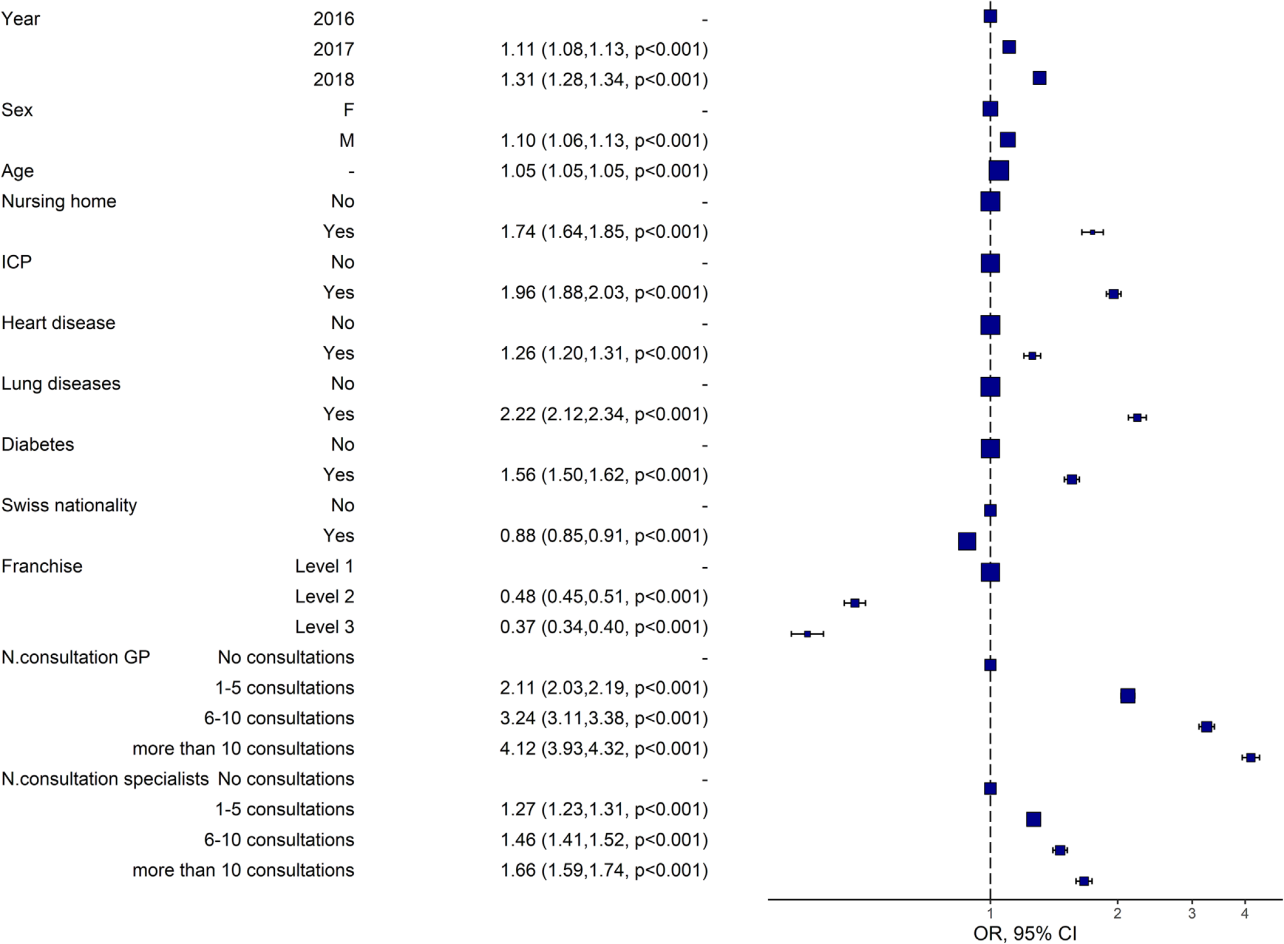
Multivariable regression analysis. Mixed logistic model with random effects at the patient level. Deductible Level 1: ≤500, Level 2: 501–1500, and Level 3: 1501–2500 (Level 3) CHF, 1 CHF = 1.0824 USD (01/01/2023). CI, confidence interval; F, female; GP, general practitioner; ICP, immunocompromised patients (cancer, human immunodeficiency viruses, autoimmune disease or drug immunosuppression, lymphoma, leukemia or myeloma, and transplantations); M, male; *N*, number; OR, odds ratio.

The highest odds of being vaccinated were found in patients with high number (>10) of GP consultations (OR: 4.12, 95% CI: 3.93–4.32, *p* < 0.001) and in patients with chronic lung diseases (OR: 2.22, 95% CI: 2.12–2.34, *p* < 0.001). In contrast, being Swiss citizen (OR: 0.88, 95% CI: 0.85–0.91, *p* < 0.001) and having an insurance contract with high deductible (OR: 0.37, 95% CI: 0.34–0.40, *p* < 0.001) were associated with decreased odds of being vaccinated (Figure 1).

### 3.3 Identification of chronic diseases by trigger type

**FIGURE 2:**
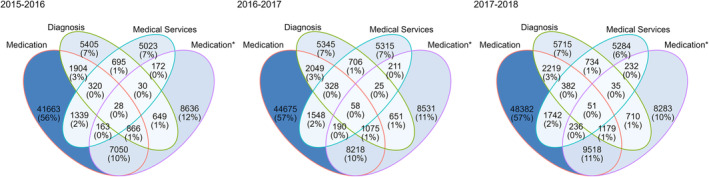
Venn diagram of individual patients identified by triggers in each season (2015/2016–2017/2018). The figure shows the absolute and relative numbers, count (%), of individual patients with chronic diseases identified by the four used trigger types. *Medication trigger based on Anatomical Therapeutic Chemical codes (instead of pharmaceutical cost group).

The relative importance of the used triggers to identify patients with a specific chronic disease is shown in Figures 2 and S1. PCG medication‐based trigger identified most of the patients with a chronic disease (72.1%–75.2% in all three analyzed seasons). ATC medication‐based trigger (23.8%–24.0%), the diagnosis‐based trigger (13.0%–13.4%), and the medical services‐based trigger (10.3%–10.6%) identified fewer patients. At the individual chronic disease level, there was more heterogeneity regarding patient identification with a single trigger.

### 3.4 Models to determine chronic disease status and dependent VURs

We identified less patients with specific chronic diseases using triggers of the given season only (Model 1) compared with the approach using triggers in the given and previous seasons (Model 2). However, agreement between the two models was almost perfect, above 90%, in many cases and strong, above 80%, in almost all risk groups (Table S2). The relative relevance of the different triggers in identifying chronic diseases, in season 2017/2018, with Model 2 is presented in Figure S2. We found, compared with Model 1, that less patients were identified with the medication‐based triggers and that more patients were identified with the diagnosis trigger. We found no significantly different VURs in patients with chronic diseases identified by the two models (24.0% vs. 24.4%, *p* = 0.07). An overview of all VURs in all subgroups is presented in Table S3.

## 4 DISCUSSION

In this retrospective study, we determined VUR among at‐risk patients, consisting of patients aged ≥65 years or adults ≥18 years with a chronic disease. We found overall VUR of about 20%. The majority of patients with chronic diseases could be identified with a trigger based on medication claims. We found no clinical significant difference in determining VUR among patients with chronic diseases in the model using trigger in the given and previous seasons (Model 2) compared with the model using only trigger in the given influenza season (Model 1).

### 4.1 Vaccination uptake

VUR was low among all analyzed groups. The highest VUR was found in patients ≥65 of age having a chronic disease, in patients with immunosuppressive conditions, and in patients living in nursing homes. In none of the subgroups analyzed, we found VUR even close to the WHO target of 75%.

Our VURs were considerably lower compared with those of the national surveillance. The Federal Office of Public Health (FOPH) reported VURs of 32%–38% in patients ≥65 and of 25%–30% in patients with chronic diseases.^31^, ^32^ Several factors may explain this difference. First, influenza VUR depends on the data source^33^, ^34^ and validation studies suggest that VUR based on self‐reported vaccination status overestimates the true VUR.^15^, ^16^ Second, vaccinations done in pharmacies are usually not covered by the basic health insurance and are paid out‐of‐pocket and these claims are seldom forwarded to the insurance company. In addition, patients might be vaccinated at work. The employer usually covers vaccination at work. Although only a small proportion of vaccinations take place in pharmacies or at work,^13^, ^35^ the effect of these vaccinations on our calculation cannot be determined. Last, VURs vary regionally^11^, ^13^ and the overrepresentation of patients from the French‐speaking part of Switzerland^36^ might contribute to the observed difference. Interestingly, VURs in patients with chronic diseases were more similar to the VURs reported by the FOPH.^31^, ^32^ Patients with chronic diseases, compared with patients without chronic diseases, are more likely to be vaccinated at GP practices^13^ and these vaccinations are recorded by the insurance company.

Apart from the national surveillance, there is only few and heterogeneous comparative national literature. One study reported VUR of about 35%, similar to the national surveillance, among patients aged 60–85 years.^13^ Two studies on diabetes patients reported VURs between about 10%^37^ and 60%.^38^ Twice as high VURs are reported in chronic obstructive pulmonary disease (COPD) patients,^39^and VUR in Swiss nursing homes was up to 63%.^40^ At the international level, our findings are similar to VUR reported in Germany or Italy.^41^, ^42^, ^43^

### 4.2 Factors associated with vaccine uptake

Our findings that patients with chronic diseases, with advanced age, or who are regularly followed up by physicians are more commonly vaccinated were in line with previous research.^9^, ^13^, ^41^, ^44^, ^45^, ^46^, ^47^ Interestingly, non‐Swiss residents were more likely to be vaccinated compared with Swiss residents unlike reported in a national health survey.^44^ One explanation could be the different vaccination strategies abroad^48^ and may reflect the inferior VUR in Switzerland compared with other European countries.^8^ We found that high deductibles were associated with lower VUR. High deductible health care plans seem to discourage patients from regular physician visits and forgoing of health care services.^49^, ^50^ The fact that vaccinations, which are actually covered by the insurance, may not be given due to high deductibles is of great concern. These findings highlight the need for efforts to better inform the population about the importance of vaccination and the cost coverage by the mandatory basic health insurance once the deductible level has been reached.

### 4.3 Methodological aspects of using claims data

PCG‐based medication trigger was the most promising, as we identified almost three quarters of all chronic diseases patients with this trigger and because PCGs are available as part of the cost equalization process in Switzerland. However, medication data can be used to infer chronic diseases^51^, ^52^, ^53^ even in countries that do not use the PCG system.

We used two models to determine whether considering triggers from previous seasons in the identification of chronic diseases (Model 2) differs from considering triggers from the current season only (Model 1). With the more complex Model 2, more patients with chronic diseases were identified and the diagnosis‐ and medical service‐based triggers had a greater relative importance. However, the VUR remained similar for almost all patient groups with not clinically relevant (even if statistically significant) differences in some subgroups.

In conclusion, conducting basic vaccination surveillance taking all available data into account for chronic diseases identification showed no advantages compared with the easier‐to‐perform and more cost‐effective season‐only‐based approach as performed in Model 1. However, when the focus is on longitudinal tracking of individuals, or if more detailed analysis on specific patient subgroups is needed, a longitudinal approach as in our Model 2 may be superior.^54^

### 4.4 Importance of the study and recommendations

Seasonal influenza causes a high public health burden.^55^ To reduce the vaccine‐preventable burden of disease, stakeholders should optimize efforts to identify unvaccinated patients and to increase the national VUR. In this context, surveillance of VUR has an important role to play, as it allows to monitor and to adjust efforts made. This study, as far as we know, is the first to have determined influenza VUR within at‐risk patients in Switzerland, using claims data and a search strategy designed to detect as many as possible patients with chronic diseases. Both identification of at‐risk patients and the VUR were free of self‐reporting biases. Similar studies have primarily used ICD‐10 codes to screen for chronic conditions.^41^, ^42^ Due to the unavailability of ICD codes in Swiss claims data, we could not perform this analysis and compare the different approach. This study demonstrated the potential of claims data and triggers to conduct influenza vaccination surveillance. Surveillance based on claims data could be supplementary to national surveillance, providing more detailed information especially for at‐risk groups.

### 4.5 Limitations

Our study has some limitations. First, our search strategy, designed to detect as many as possible patients with chronic diseases of interest, may led to false positive inclusions. This concerns, in particular, patients with immunosuppression drugs, as immunosuppressive therapies may be time limited or dose adjusted during the course of therapy. In addition, we were unaware of exclusion criteria such as medical reasons to avoid the vaccination, for example, allergies to vaccine components. Second, as mentioned above, there is a low risk of underestimating the true VUR due to vaccinations in pharmacies and at work. Third, the two models identifying patients with chronic diseases and the consecutive comparison of VUR are based on only three consecutive influenza seasons data. Whether the VUR would differ over longer observation periods needs further research. Fourth, although we could identify factors associated with vaccination, we could not adjust them for underlying psychological frameworks related to the vaccination willingness, which might confound these associations. Finally, chronic diseases included are derived from the national immunization schedule and therefore not exhaustive, as in other countries, the vaccination recommendations differ.^8^

## 5 CONCLUSIONS

Our study highlighted the need for efforts to increase national influenza vaccination uptake among at‐risk patients. We identified several factors associated with vaccination status, which may contribute to adjust national vaccination campaigns, both for the general population and for specific risk groups. Finally, our results should encourage the use of routinely available claims data to conduct basic vaccination surveillance in order to support the national surveillance.

## AUTHOR CONTRIBUTIONS

**Andreas Plate:** Conceptualization; methodology; project administration; supervision; validation; writing—original draft; writing—review and editing. **Christophe Bagnoud:** Data curation; validation; writing—review and editing. **Thomas Rosemann:** Conceptualization; resources; writing—review and editing. **Oliver Senn:** Conceptualization; methodology; writing—review and editing. **Stefania Di Gangi:** Conceptualization; data curation; formal analysis; methodology; validation; visualization; writing—review and editing.

## CONFLICT OF INTEREST STATEMENT

The authors declare no conflicts of interest.

## ETHICS APPROVAL STATEMENT

Research using anonymized and aggregated data from health care insurances did not fall under the Swiss Federal Act on Research involving Human Beings (Human Research Act) and thus no ethics approval was needed.

## Supporting information


**Table S1:** Overview of all used triggers to identify chronic diseases.
**Table S2:** Comparison of identification of chronic disease by triggers in the given season only (Model 1) and by triggers in the given and previous season(s) (Model 2).
**Table S3:** Comparison of vaccine uptake rates between the model using triggers in the given season only (Model 1) and the model using triggers in the given and previous season(s) (Model 2).
**Figure S1:** Overview of individual patients identified by a given trigger and by comorbidity season 2017/2018.
**Figure S2:** Venn diagram of individual patients identified by triggers (model 2), (seasons 2015/2016–2017/2018).

## Data Availability

Research data are not shared.
