# Supplementary material for: Influenza vaccination uptake among at‐risk patients in Switzerland—The potential of national claims data for surveillance
Source: Influenza Other Respir Viruses. 2023 Oct 13;17(10):e13206. doi: 10.1111/irv.13206 (PMC10570900; doi:10.1111/irv.13206)

**Influenza vaccination uptake among at-risk patients in Switzerland – the potential of national claims data for surveillance**

Andreas Plate, Christophe Bagnoud, Thomas Rosemann, Oliver Senn, Stefania Di Gangi

- **Supplemental table 1**: Overview of all used triggers to identify chronic diseases
- **Supplemental table 2**: Comparison of identification of chronic disease by triggers in the given season only (Model 1) and by triggers in the given and previous season(s) (Model 2)
- **Supplemental table 3**: Comparison of vaccine uptake rates between the model using triggers in the given season only (Model 1) and the model using triggers in the given and previous season(s) (Model 2)
- **Supplemental figure 1**: Overview of individual patients identified by a given trigger and by comorbidity season 2017/2018
- **Supplemental figure 2**: Venn diagram of individual patients identified by triggers (model 2), (seasons 2015/2016 – 2017/2018)

Supplemental table 1: Overview of all used triggers to identify chronic diseases.

| Comorbidity | Trigger | | | |
| --- | --- | --- | --- | --- |
|  | PCG^1^ | DRG^2^ | TARMED^3^ | ATC^4^ |
| Chronic heart disease | CAR, HYP | F01A, F01B, F01C, F01D, F01E, F01F, F02A, F02B, F03A, F03B, F03C, F03D, F03E, F06A, F06B, F06C, F06D, F12A, F12B, F12C, F12D, F12E, F12F, F17A, F17B, F18A, F18B, F24A, F24B, F24C, F24D, F24E, F24F, F60A, F60B, F62A, F62B, F62C, F62D, F66A, F66B, F67A, F67B, F68A, F68B, F69A, F69B, F98A, F98B, F98C | 17.1110, 17.1210, 17.1510, 17.1520, 17.1540, 17.1560, 17.1570, 17.1590, 17.1600, 17.1610, 17.1620, 17.1630, 17.1640, 17.1650, 17.1660, 18.0110, 18.0160, 18.0170, 18.0210, 18.0230, 18.0240, 18.0250, 18.0260, 18.0300, 18.0310, 18.0330, 18.0340 |  |
| Chronic lung disease | AST, COP, ZFP | E60A, E60B, E65A, E65B, E65C, E65D, E65E |  |  |
| Chronic liver disease |  | H09A, H09B, H09C, H60A, H60B, H60C | 19.0210 + (19.0260 or 19.0270 or 19.0280 or 19.0290), 19.0410 + (19.0460 or 19.0470 or 19.0480 or 19.0490) |  |
| Chronic kidney disease | NIE | L60A, L60B, L60C, L60D, L60E |  |  |
| Cerebral disease | MSK, ZNS | B67A, B67B, B68A, B68B, B68C, B69A, B69B, B69C, B69D, B70A, B70B, B70C, B70D, B70E, B70F, B70G, B70J, B70K |  |  |
| Diabetes mellitus | DM1, DM2, DM2+ |  |  |  |
| Lymphoma, leukemia, myeloma |  | R60A, R60B, R60C, R60D, R61A, R61B, R61C, R61D, R63A, R63B, R63C, R63D, R63E |  |  |
| Transplantations | TRA | A01A, A01B, A02Z, A04A, A04B, A05A, A05B, A60A, A60B, A60C | 21.0960 |  |
| Autoimmune disease or drug immunosuppression | AIK, RHE |  |  | L01, L04 |
| HIV | HIV | S01Z, S62Z, S63A, S63B, S65Z |  |  |
| Cancer | KRE, KRK | A93A, A93B, A93C, A93D, B16Z, D35Z, D60A, D60B, E03Z, E05B, E08A, E08B, E71A, E71B, G16Z, G17A, G19A, G29B, G60A, G60B, H61A, H61B, I54A, I54B, I65A, I65B, J01A, J01B, J06A, J06B, J18A, J18B, J23A, J23B, J62A, J62B, K15A, K15B, L12Z, L62A, L62B, M09A, M09B, M10Z, M60A, M60B, N01A, N01B, N01C, N16Z, N60A, N60B, R01A, R01B, R01C, R01D, R01E, R50A, R50B, R50C, R62A, R62B, R62C, R65A, R65B, R65C | 04.0320, 04.0330, 04.0340, 04.0350, 05.1220, 05.1240, 05.1250, 05.1260, 05.1270, 05.1280, 05.1290, 05.1300, 05.1310, 05.1320, 05.1330, 05.1340, 05.1350, 05.1360, 05.1370, 05.1380, 05.1390, 05.1400, 05.1410, 05.1420, 05.1430, 05.1440, 07.1240, 07.1260, 10.1230, 10.1240, 11.0270, 11.0300, 11.0310, 11.0320, 11.0800, 11.0810,  12.0330,  12.0360,  12.0370,  12.0390,  14.0360 + 14.0370, 16.0150, 16.0250, 16.0840 + 16.0940, 21.0510 + 21.0600, 21.0920, 21.0970, 22.0360 + 22.0470, 23.0140, 23.0150, 23.0190, 23.0200, 23.0210, 24.0560, 24.0570, 24.1420, 24.1440, 24.1820, 24.2270, 24.2280, 24.2640, 24.4130, 24.8570, 26.0030, 26.0050, 26.0230, 26.0340, 26.0350, 26.0420, 26.0450, 32. |  |
| 1: medication based trigger: Pharmaceutical Cost Group, PCG;  2: diagnosis based trigger: Diagnosis-related group, DRG  3: medical services based trigger: Procedure codes, TARMED  4: medication based trigger: WHO Anatomical Therapeutic Chemical Code, ATC | | | | |

Supplemental table 2: Comparison of identification of chronic disease by triggers in the given season only (Model 1) and by triggers in the given and previous season(s) (Model 2). Cohen’s Kappa (κ) was reported to measure the agreement between the two methods. We considered agreement as strong, and for our purposes acceptable, if Cohen’s κ coefficient was in the range 0.80-0.90; above 0.90 meant almost perfect agreement.

| Influenza season | | 2015/2016 | 2016/2017 | |  | 2017/2018 | |  |
| --- | --- | --- | --- | --- | --- | --- | --- | --- |
|  | |  | Model 1 | Model 2 | κ | Model 1 | Model 2 | κ |
|  | Types of risk | | | | | | |  |
| Chronic disease independent of age | | 73,943 ( 44.1) | 78,925 ( 44.5) | 86,258 (48.7) | 0.92 | 84,702 ( 46.4) | 96,491 ( 52.9) | 0.87 |
| Age ≥ 65 without chronic disease | | 93,664 ( 55.9) | 98,247 ( 55.5) | 90,925 ( 51.3) | 0.93 | 97,727 ( 53.6) | 85,955 ( 47.1) | 0.90 |
| Chronic disease and age ≥ 65 | | 35,001 ( 20.9) | 37,730 ( 21.3) | 44776 ( 25.3) |  | 40,831 ( 22.4) | 52,171 ( 28.6) |  |
| Chronic disease and age < 65 | | 38,942 ( 23.2) | 41,195 ( 23.3) | 41482 ( 23.4) |  | 43,871 ( 24.0) | 44,320 ( 24.3) |  |
|  | Chronic diseases | | | | | | |  |
| Chronic heart disease | | 12,476 (7.4) | 13,175 (7.4) | 16,322 (9.2) | 0.88 | 13,665 (7.5) | 19,055 (10.4) | 0.82 |
| Chronic lung disease | | 13,751 (8.2) | 14,962 (8.4) | 16,519 (9.3) | 0.95 | 15,270 (8.4) | 17,920 (9.8) | 0.91 |
| Diabetes | | 24,188 (14.4) | 26,164 (14.7) | 27,867 (15.7) | 0.96 | 30,257 (16.6) | 32,546 (17.8) | 0.96 |
| Cancer | | 7167 ( 4.3) | 7674 (4.3) | 9791 (5.5) | 0.87 | 8453 (4.6) | 12,116 (6.6) | 0.81 |
| Chronic kidney disease | | 529 (0.3) | 568 (0.3) | 832 (0.5) | 0.81 | 559 (0.3) | 1050 (0.6) | 0.69 |
| HIV † | | 2114 (1.3) | 2225 (1.3) | 2265 (1.3) | 0.99 | 2214 (1.2) | 2282 (1.2) | 0.98 |
| Cerebral disease | | 3831 (2.3) | 4073 (2.3) | 5430 (3.1) | 0.85 | 3929 (2.2) | 6620 (3.6) | 0.74 |
| Chronic liver disease | | 210 (0.1) | 166 (0.1) | 281 (0.2) | 0.74 | 312 (0.2) | 482 (0.3) | 0.79 |
| Autoimmune disease or drug immunosuppression | | 17,826 (10.6) | 19,206 (10.8) | 21,898 (12.4) | 0.93 | 20,493 (11.2) | 25,162 (13.8) | 0.88 |
| Lymphoma, leukemia or myeloma | | 186 (0.1) | 184 (0.1) | 270 (0.2) | 0.81 | 299 (0.2) | 443 (0.2) | 0.81 |
| Transplantations | | 821 (0.5) | 897 (0.5) | 1026 (0.6) | 0.93 | 882 (0.5) | 1119 (0.6) | 0.88 |

†: HIV: human immunodeficiency viruses

Supplemental table 3: Comparison of vaccine uptake rates between the model using triggers in the given season only (Model 1) and the model using triggers in the given and previous season(s) (Model 2); p-values (p) from chi-square test for two proportions, two sided, were reported.

| Influenza season | | 2015/2016 | 2016/2017 | |  | 2017/2018 | |  |
| --- | --- | --- | --- | --- | --- | --- | --- | --- |
|  | |  | Model 1 | Model 2 | p | Model 1 | Model 2 | p |
|  | Type of risk | | | | | | |  |
| Chronic disease independent of age | | 16,403 (22.2) | 18,119 (23.0) | 20,050 (23.2) | 0.169 | 20,349 (24.0) | 23,532 (24.4) | 0.072 |
| Age ≥ 65 without chronic disease | | 14,412(15.4) | 15,232 (15.5) | 13,301 (14.6) | <0.001 | 15,729 (16.1) | 12,549 (14.6) | <0.001 |
| Chronic disease and age ≥ 65 | | 10,856 (31.0) | 12,113 (32.1) | 14,006 (31.3) | 0.011 | 13,817 (33.8) | 16,941 (32.5) | <0.001 |
| Chronic disease and age < 65 | | 5547 (14.2) | 6006 (14.6) | 6044 (14.9) | 0.978 | 6532 (14.9) | 6591 (14.9) | 0.949 |
|  | Chronic diseases | | | | | | |  |
| Chronic heart disease | | 3489 (28.0) | 3735 (28.3) | 4653 (28.5) | 0.774 | 4051 (29.6) | 5604 (29.4) | 0.654 |
| Chronic lung disease | | 3460 (25.2) | 3884 (26.0) | 4336 (26.2) | 0.568 | 4234 (27.7) | 5074 (28.3) | 0.240 |
| Diabetes | | 5087 (21.0) | 5645 (21.6) | 6122 (22.0) | 0.273 | 6755 (22.3) | 7457 (22.9) | 0.081 |
| Cancer | | 1394 (19.5) | 1662 (21.7) | 2180 (22.3) | 0.345 | 1978 (23.4) | 2987 (24.7) | 0.040 |
| Chronic kidney disease | | 142 (26.8) | 183 (32.2) | 269 (32.3) | 1.000 | 183 (32.7) | 355 (33.8) | 0.705 |
| HIV † | | 830 (39.3) | 865 (38.9) | 881 (38.9) | 1.000 | 802 (36.2) | 830 (36.4) | 0.943 |
| Cerebral disease | | 577 (15.1) | 680 (16.7) | 1055 (19.4) | 0.001 | 720 (18.3) | 1477 (22.3) | <0.001 |
| Chronic liver disease | | 31 (14.8) | 28 (16.9) | 53 (18.9) | 0.688 | 56 (17.9) | 94 (19.5) | 0.650 |
| Autoimmune disease or drug immunosuppression | | 3543 (19.9) | 4027 (21.0) | 4656 (21.3) | 0.472 | 4675 (22.8) | 5891 (23.4) | 0.134 |
| Lymphoma, leukemia or myeloma | | 41 (22.0) | 49 (26.6) | 78 (28.9) | 0.675 | 77 (25.8) | 135 (30.5) | 0.189 |
| Transplantations | | 317 (38.6) | 318 (35.5) | 370 (36.1) | 0.817 | 316 (35.8) | 403 (36.0) | 0.968 |

†:HIV: human immunodeficiency viruses

Supplemental figure 1: Overview of individual patients identified by a given trigger and by comorbidity season 2017/2018


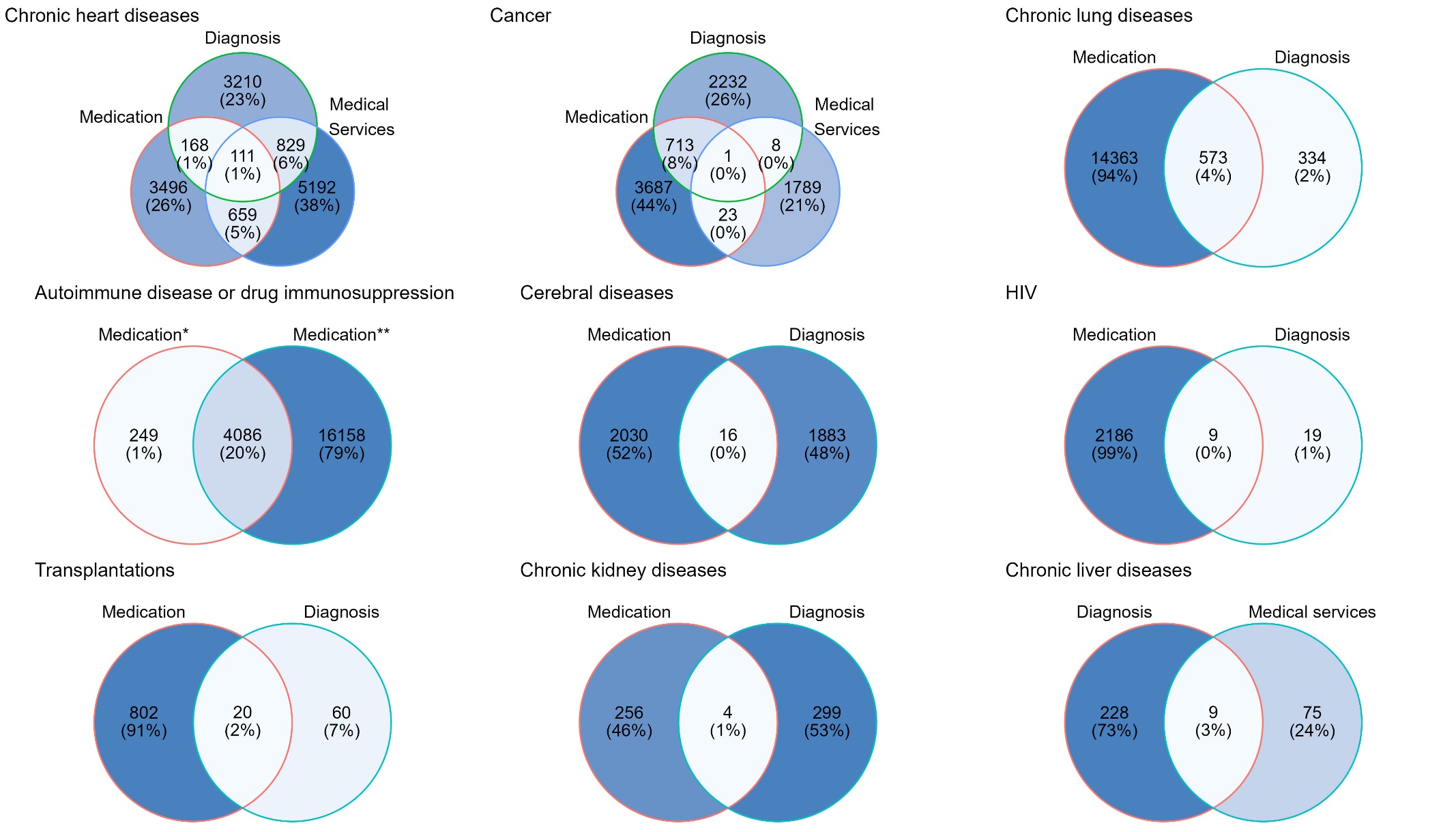


Supplemental figure 1 shows the absolute and relative numbers, count(%), of individual patients with specific chronic diseases identified by the different used trigger types. For better readability, the sizes of the circles are independent of the proportions shown. Patients with diabetes mellitus (PCG trigger) and lymphoma, leukemia or myeloma (DRG trigger) were only identified by one trigger type and not shown in this figure.*: medication trigger based on ATC codes (instead of PCG).

Supplemental figure 2: Venn diagram of individual patients identified by triggers (model 2), (seasons 2015/2016 – 2017/2018). *: medication based on ATC trigger.


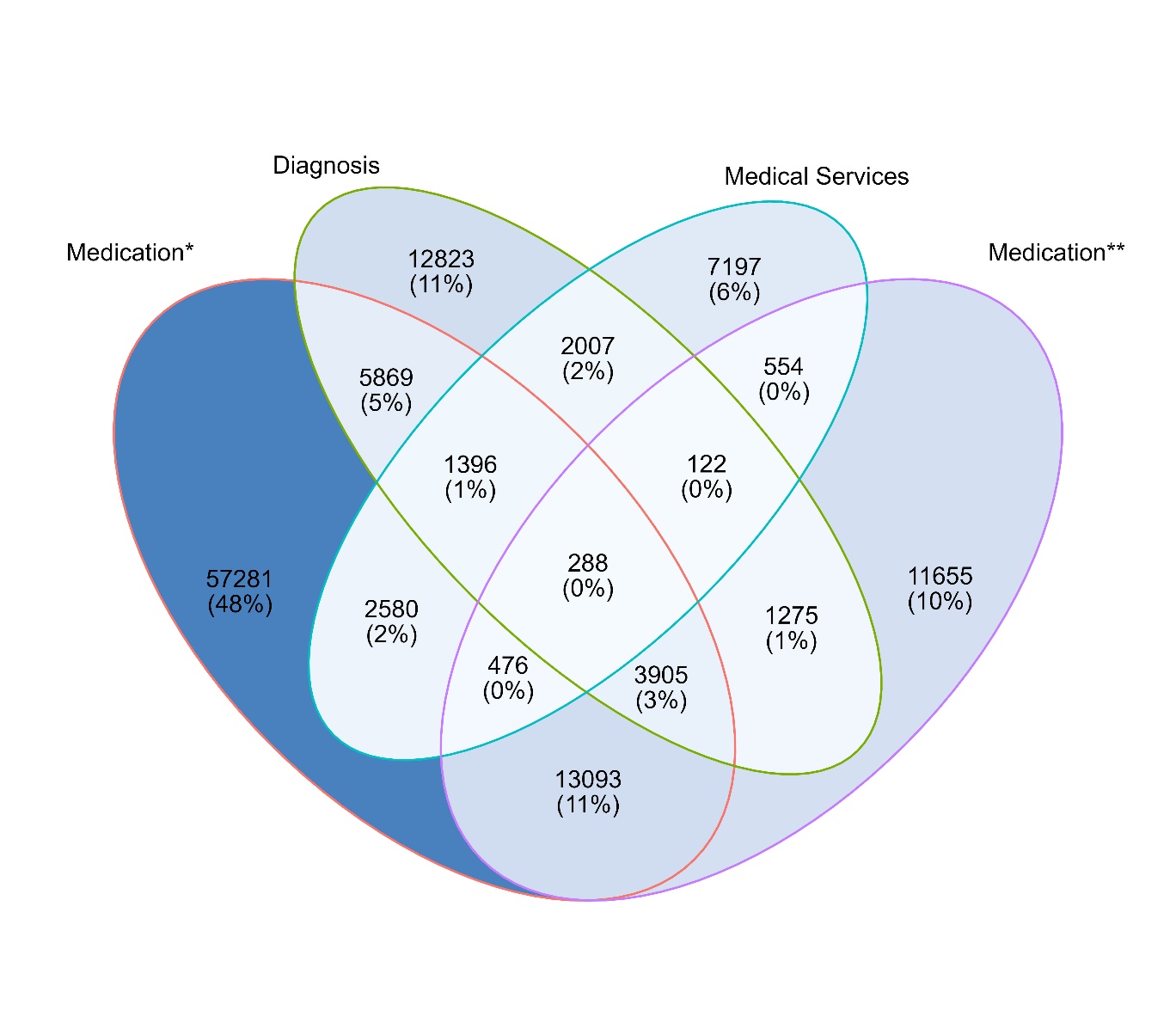

Supplement: Supplementary file 1 — Table S1: Overview of all used triggers to identify chronic diseases. Table S2: Comparison of identification of chronic disease by triggers in the given season only (Model 1) and by triggers in the given and previous season(s) (Model 2). Table S3: Comparison of vaccine uptake rates between the model using triggers in the given season only (Model 1) and the model using triggers in the given and previous season(s) (Model 2). Figure S1: Overview of individual patients identified by a given trigger and by comorbidity season 2017/2018. Figure S2: Venn diagram of individual patients identified by triggers (model 2), (seasons 2015/2016–2017/2018). [file IRV-17-e13206-s001.docx]
